# TGF-β/SMAD Pathway Is Modulated by miR-26b-5p: Another Piece in the Puzzle of Chronic Lymphocytic Leukemia Progression

**DOI:** 10.3390/cancers14071676

**Published:** 2022-03-25

**Authors:** Maria Elena Marquez, Sandra Sernbo, Eugenia Payque, Rita Uria, Juan Pablo Tosar, Juliana Querol, Catalina Berca, Angimar Uriepero, Daniel Prieto, Diego Alvarez-Saravia, Carolina Oliver, Victoria Irigoin, Gimena Dos Santos, Cecilia Guillermo, Ana Inés Landoni, Marcelo Navarrete, Florencia Palacios, Pablo Oppezzo

**Affiliations:** 1 Research Laboratory on Chronic Lymphocytic Leukemia, Institut Pasteur de Montevideo, Montevideo 11400, Uruguay; mariamarquez@pasteur.edu.uy (M.E.M.); sandrasernbo@yahoo.se (S.S.); eugeniapayque@gmail.com (E.P.); ritauria@gmail.com (R.U.); juliana.querol@gmail.com (J.Q.); catalina.berca8@gmail.com (C.B.); angimar.uriepero@gmail.com (A.U.); dprieto@pasteur.edu.uy (D.P.); palacios@pasteur.edu.uy (F.P.); 2 Nuclear Research Center, Analytical Bichemistry Unit, Faculty of Science, Universidad de la República, Montevideo 11400, Uruguay; jptosar@pasteur.edu.uy; 3 Functional Genomics Unit, Institut Pasteur de Montevideo, Montevideo 11400, Uruguay; 4 Department of Computer Engineering, University of Magallanes, Punta Arenas 6210005, Chile; diego.alvarez@umag.cl; 5 Hospital de Clínicas, Cátedra de Hematología, Montevideo 11600, Uruguay; carolinaoliver80@gmail.com (C.O.); vicirigoin19@gmail.com (V.I.); gimena2santos@gmail.com (G.D.S.); ceciliaguillermo63@gmail.com (C.G.); 6 Hospital Maciel, ASSE, Servicio de Hematología, Montevideo 11000, Uruguay; ailandoni@yahoo.com; 7 Laboratory of Molecular Medicine, School of Medicine, University of Magallanes, Punta Arenas 6210005, Chile; marcelo.navarrete@umag.cl

**Keywords:** chronic lymphocytic leukemia, microenvironment, microRNAs, TGF-β/SMAD pathway

## Abstract

**Simple Summary:**

TGF-β is a key immunoregulatory pathway that can limit the proliferation of B-lymphocytes. Chronic lymphocytic leukemia (CLL) has been historically conceptualized as a neoplasm characterized by accumulation of mature B cells escaping programmed cell death and undergoing cell-cycle arrest in the G0/G1 phase. However, new evidence indicates that tumor expansion is in fact a dynamic process in which cell proliferation also plays an important role. In general, cancers progress by the emergence of subclones with genomic aberrations distinct from the initial tumor. Often, these subclones are selected for advantages in cell survival and/or growth. Here, we provide novel evidence to explain, at least in part, the origins of CLL progression in a subgroup of patients with a poor clinical outcome. In this cohort, the immunoregulatory pathway TGF-β/SMAD is modulated by miR-26b-5p and the impairment of this axis bypasses cell cycle arrest in CLL cells facilitating disease progression.

**Abstract:**

Clinical and molecular heterogeneity are hallmarks of chronic lymphocytic leukemia (CLL), a neoplasm characterized by accumulation of mature and clonal long-lived CD5 + B-lymphocytes. Mutational status of the IgHV gene of leukemic clones is a powerful prognostic tool in CLL, and it is well established that unmutated CLLs (U-CLLs) have worse evolution than mutated cases. Nevertheless, progression and treatment requirement of patients can evolve independently from the mutational status. Microenvironment signaling or epigenetic changes partially explain this different behavior. Thus, we think that detailed characterization of the miRNAs landscape from patients with different clinical evolution could facilitate the understanding of this heterogeneity. Since miRNAs are key players in leukemia pathogenesis and evolution, we aim to better characterize different CLL behaviors by comparing the miRNome of clinically progressive U-CLLs vs. stable U-CLLs. Our data show up-regulation of miR-26b-5p, miR-106b-5p, and miR-142-5p in progressive cases and indicate a key role for miR-26b-5p during CLL progression. Specifically, up-regulation of miR-26b-5p in CLL cells blocks TGF-β/SMAD pathway by down-modulation of SMAD-4, resulting in lower expression of p21^−Cip1^ kinase inhibitor and higher expression of c-Myc oncogene. This work describes a new molecular mechanism linking CLL progression with TGF-β modulation and proposes an alternative strategy to explore in CLL therapy.

## 1. Introduction

Chronic lymphocytic leukemia (CLL) is the most common leukemia in the Western world [1]. This disease is characterized by the clonal proliferation and accumulation of mature, long-lived CD5 + B-lymphocytes in peripheral blood (PB), bone marrow (BM), lymph nodes (LN) and the spleen [2]. The clinical evolution of the patients is highly heterogeneous. One-third is diagnosed as indolent and never requires treatment; in another third, an initial indolent phase is followed by progression of the disease; and the remaining third has aggressive CLL and needs immediate treatment [3]. Additionally, the mutational profile of HV immunoglobulin genes (IgHV) divides patients into two categories [4] that differ dramatically in prognosis [5,6]. Patients expressing mutated IgHV (M-CLL) develop a more indolent disease, whereas IgHV unmutated patients (U-CLL) display the worst prognosis. Despite IgHV status being one of the most relevant prognostic factors [7], M-CLL and U-CLL often exhibit different clinical outcomes within each subgroup, and the reasons of this diversity still remain controversial. Indeed, the discovery of new prognostic markers could help to identify and better explain the origins of CLL progression, one of the most key and unsolved issues in CLL biology.

Oncogenic hallmarks of cancer progression such as genome instability and mutations, deregulated cellular energetics, avoiding immune destruction, and tumor-promoting inflammation [8] should be considered in CLL progression [9]. In fact, microenvironment interactions and/or epigenetic changes in tumor cells are at the origins of the CLL heterogeneity supporting the different clinical evolution profiles [10]. Among the multiple variables that contribute to this, the microRNA (miRNA) landscape of the tumor cell emerges as a central player in the pathogenesis and evolution of CLL [11,12,13]. Several miRNA signatures [14], as well as specific miRNAs, distinguish between different CLL subgroups [15] and show diagnostic or prognostic value [16]. miRNAs have been involved in CLL progression [12], linked to therapy resistance [16], or linked to different B cell activation pathways [17,18,19,20], but the molecular basis behind the function of most of these molecules remains poorly understood in CLL.

In this work, we specifically focus on those patients with unmutated IgHV status and different clinical outcomes. We performed miRNome analysis from eleven CLL cases, (five clinically stable U-CLLs and six clinically progressive U-CLLs). miRNome profiles were compared between these two subgroups; subsequently, the up-regulated miRNAs in patients with the poorest outcomes were selected and validated by quantitative PCR (q-PCR) in an additional cohort of 15 cases. TGF-β signaling maintains tissue homeostasis and prevents incipient tumors from progressing down the path to malignancy [21,22]. This pathway regulates not only cellular proliferation, differentiation, survival, and adhesion but also the cellular microenvironment [23]. Several works suggest that TGF-β inhibition promotes leukemic transformation [24,25], and, specifically in CLL, this has been postulated [26,27,28,29], but the molecular mechanisms that regulate this pathway during CLL progression remain unknown.

Here, we provide additional evidence supporting the view that the loss of sensitivity to TGF-β pathway contributes to the clinical and biological progression of CLL [27,30]. We describe a key role for the miR-26b-5p down-modulating the TGF-β/SMAD axis in progressive U-CLL. Our results show that miR-26b-5p is up-regulated in this subgroup, whereas SMAD-4 protein expression is decreased and mostly excluded from the nuclei of tumor cells. The kinase inhibitor p21^−Cip1^ and the oncogene c-Myc are the two target genes by which TGF-β maintains tissue homeostasis, inhibiting the progression of tumor cells to the cell cycle phase G1 [25]. Supporting the idea about the existence of an impairment in TGF-β/SMAD signaling in progressive U-CLL cases, we found low expression of the kinase inhibitor p21^−Cip1^ and high expression of c-Myc compared with stable U-CLL. Finally, by down-regulation of miR-26b-5p in progressive U-CLL cases, we corroborate up-regulation of SMAD-4 and p21^−Cip1^ proteins and down-modulation of c-Myc gene in the transfected CLL cells. These results propose for the first time a specific role for miR-26b-5p in the TGF-β pathway in CLL, suggesting that the leukemic clone in progressive cases could acquire tumor fitness advantages by regulating the homeostatic control of TGF-β/SMAD pathway during CLL progression.

## 2. Materials and Methods

### 2.1. CLL Cohort

Peripheral CLL cells were isolated from CLL patients by density centrifugation (Ficoll-Paque, HealthCare Life Sciences). B cells were isolated by magnetic anti-CD19 Microbeads separation (*Miltenyi Biotec*) to obtain purity of ≥95% of CD5 + 19 + cells (evaluated by flow cytometry). CLL patients were segregated into two groups of unmutated patients: Stable patients (defined by: Binet stage A, lymphocyte doubling time >1 year, absence of Lipoprotein lipase (LPL) and activation-induced cytidine deaminase (AID) mRNA expression in PB, and no treatment requirement after a 4-year follow-up) and Progressive patients (defined by: Binet stage B/C, lymphocyte doubling time <6 months, expression of AID and LPL mRNAs in PB, and treatment requirement before 3 years). Fresh samples were collected in a prospective manner from patients with CLL defined by the International Workshop on Chronic Lymphocytic Leukemia (IwCLL) criteria. The procedures were according to the guidelines of the medical ethics board of the Hospital Maciel (ethical approval code C.E.I.H.M_24/04/19). Written permissions for the use of the samples were obtained from the patients according to the ethical regulations from Uruguay and the Helsinki Declaration.

### 2.2. microRNAome Analysis

miRNAs were isolated from B cells obtained from eleven CLL cases, 5 stable patients and 6 progressive patients, using the mirVana isolation kit (Invitrogen Cat. AM1561). A small RNA library was generated using the Illumina TruseqTM Small RNA Preparation kit according to manufacturer guides; the purified cDNA library was used for cluster generation on Illumina’s Cluster Station and then sequenced on Illumina GAIIx. A proprietary pipeline script, ACGT101-miR v4.2 (LC Sciences), was used for sequencing data analysis. For comparison analysis, raw reads of each sample were combined for mapping tracking the copy number of reads during mapping. The significance of differential expression was calculated by two-tailed T-Test (Supplementary Materials Table S1).

After obtaining a list of differentially expressed miRNAs, 8 of them were selected and validated by q-PCR. For miRNAs, the RT-PCR was performed using stem-loop (SL) primers, as described previously [31]. Briefly, 6 ng of small RNA was mixed with folded SL-primers at a final concentration of 10 nM and final volume of 7 μL. Then, 2 μL of these were loaded in the qPCR reaction (V = 10 μL) with 1.5 μM of forward primers and 0.7 μM of the universal reverse primers. All qPCR reactions were run on the Eco Real-time PCR System (Ilumina), using a two-step protocol, with specific miRNA primers, as specified in Supplementary Materials Table S2, using the Faststart Universal SYBR Green (Roche). U6 snRNA were used as endogenous controls for miRNA, and the relative expression was calculated as 2^−ΔΔCt^.

### 2.3. mRNA Expression Analysis

RNA extraction was performed from 1 × 10^6^ to 5 × 10^6^ PBMC from CLL patients using Trizol (Invitrogen, Carlsbad, CA, USA), and cDNA was synthesized as described in [32], using M MLV reverse transcriptase (Invitrogen) with ribonuclease inhibitor RNAsin (Promega, Madison, WI, USA). Standard and quantitative polymerase chain reaction (q-PCR) amplification was performed as described in [33]. SMAD-2, SMAD-4, SMAD-3, SMAD-7, p21^−Cip1^, KLF10, c-MYC, and GAPDH primers are provided in Supplementary Materials Table S2. Glyceraldehyde-3-phosphate dehydrogenase (GAPDH) was used as an endogenous control, whereas cDNA of MEC-1 cells were used as a reference sample. The relative expression was calculated as 2^−ΔΔCt^. All qPCR reactions were run on the QuantStudio3 Real-time PCR system (Applied Biosystems).

### 2.4. Confocal Microscopy and Quantification of Nuclear Staining

Cells previously fixed in 4% of paraformaldehyde (PFA) were applied to prepared slides and left 2 h at room temperature (RT) to adhere to slides. Cells were then blocked with PBS/3%BSA/0.1%triton for 30 min at RT and left with antibodies: α-IgM-PE (1/40) (Jackson) and α-Smad4-Alexa 647 (1/250) (sc-7966 AF647, Santa Cruz Biotechnology, Santa Cruz, CA, USA), O/N at 4 °C. After washing with PBS, cells were incubated with Hoechst 33342 for nuclear staining for 5 min. Images were captured in a Zeiss LSM 800 microscope (Zeiss, Jena, Germany) using Zen system 2.3.

### 2.5. Measurements of Surface and Intracellular Antigens by Flow Cytometry

Live cells were identified using Zombie Aqua fixable viability kit (Biolegend, San Diego, CA, USA). For surface membrane immunofluorescence, cells (0.5 × 10^6^) in FACS buffer (PBS + 2% bovine serum) were incubated with primary antibody for 20 min at 4 °C. For intracellular detection, after surface membrane staining with anti-CD19-percpCy5.5 anti-CD5-fitc (1/50) (Biolegend), cells were fixed and permeabilized (Cytofix/Cytoperm, BD Biosciences, San Jose, CA, USA) and incubated with α-Smad-4-Alexa 647 (1/25) and p21^−Cip1^ rabbit mAb (1/200) (Cell signaling) followed by goat antirabbit IgG-Cy5 (1/1000) (ThermoFisher, Waltham, MA, USA). The expression level was defined by the relative mean fluorescence intensity (MFI) or relative expression in terms of percent positive cells. Data were acquired with Attune NxT flow cytometer (Thermo Fisher Scientific) and analyzed by FlowJo 10.8 version.

### 2.6. miRNA Transfections and Sorting

PBMC from CLL patients were transfected with miRNA hairpin inhibitor hsa-miR-26b-5p (miRIDIAN, Dharmacon, Lafayette, CO, USA) or irrelevant antagomiR conjugated to Dy547 (Dharmacon). All molecules were synthesized by Thermo Fisher Scientific, Dharmacon Products. Briefly, PBMC (20 × 10^6^) were resuspended in Opti-MEM I (Invitrogen, Carlsbad, CA, USA) and transfected with 50 nM of miRNA and 5 µL/mL of lipofectamine RNAiMAX (Invitrogen, Carlsbad, CA, USA), as previously described in [33]. After 20 h, the cells were centrifuged and stained with anti-CD19-PercpCy5.5 and anti-CD5-FITC. Then, isolation by cell sorter of not transfected (Dy547-negative) and transfected (Dy547-positive) CD19 + CD5 + CLL cells was performed with the use of the MoFlo (Beckman Coulter, South Kraemer Blvd Brea, CA, USA). In all cases, purity of isolated populations was greater than 96%.

### 2.7. Statistical Analysis

Statistical significance was determined using nonparametric tests: Wilcoxon signed-rank test, Mann–Whitney test. In all cases, *p* < 0.05 was considered statistically significant, (* = *p* < 0.05, ** = *p* < 0.01, and **** = *p* < 0.0001). All analyses were performed using GraphPad Prism 7.00. GraphPad Software Inc.

### 2.8. NGS Data Availability

The sequence data are deposited in DRYAD: doi:10.5061/dryad.n2z34tmzd. Data are accessible for peer review at the following link: https://datadryad.org/stash/share/flVsy1kgg770p5Ef6vAoYLRL1SpafGnt2oricQ8X6oo, accessed on 10 February 2022.

## 3. Results

### 3.1. miRNAome Profile of U-CLL with Different Clinical Outcomes and Validation of Up-Regulated miRNAs in the Clinically Progressive Subgroup

**Figure 1:**
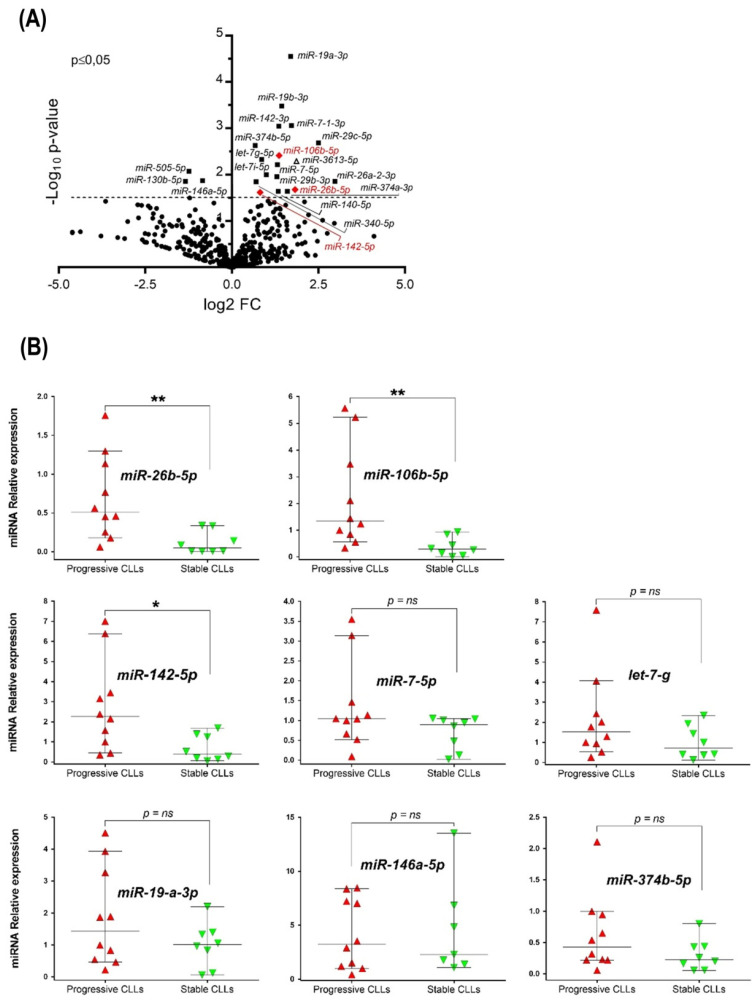
miRNAome analysis and data validation of selected miRNAs differentially expressed between progressive and stable U-CLL subgroups. (**A**) Volcano plot showing transcriptome analysis of eleven U-CLL patients: 5 stable patients and 6 progressive CLLs. Solid squares = miRNAs differentially expressed (*p* ≤ 0.05). Red diamond indicates those miRNAs validated by q-PCR (**B**) Quantitative PCR analysis validating the initial transcriptome data. An additional larger cohort with similar biologic and clinical characteristics (*n* = 15) was investigated. Mann–Whitney Unpaired test and median with 95% CI are depicted. U6 snRNA were used as endogenous quantity controls for miRNA, and the relative expression was calculated as 2^−ΔΔCt^. In all cases, *p* < 0.05 was considered statistically significant, (* = *p* < 0.05, ** = *p* < 0.01, ns = not significant).

To identify relevant miRNAs during CLL progression in U-CLL, we performed miRNome analysis from eleven CLL cases, five stable and six progressive U-CLLs. Clinical and biological characteristics of the patients in the study are provided in Table 1. Differential expression of up- and down-regulated miRNAs in progressive cases are depicted in Figure 1A (Volcano plot) and detailed in the Table 2. Our results identify 21 miRNAs differentially expressed, 3 down-regulated and 18 up-regulated in progressive cases compared with stable U-CLL, (Figure 1A and Table 2). From these 21 miRNAs, 13 were previously described in CLL, and 7 are mentioned here for the first time (Table 2). Considering these results and aiming to identify miRNAs involved in disease progression of U-CLL, we selected a final list of 8 miRNAs, (all of them involved in tumor progression, according to bibliographical data, Table 2). We validated their relative abundance by q-PCR in a larger CLL cohort of 15 additional U-CLL patients (8 progressive and 7 stable, Table 1). After this analysis, only 3 of the 8 selected miRNAs depicted statistically significant changes comparing the relative abundance between stable and progressive subgroups of the additional cohort (Figure 1B). The miR-26b-5p, miR-106b-5p, and miR-142-5p were up-regulated in the progressive cases (*p* = 0.003, *p* = 0.001, and *p* = 0.011, respectively, Mann–Whitney Unpaired test, *n* = 18). Interestingly, despite not being selected based on this characteristic; they have all been involved in the modulation of TGF-β/SMAD pathway [21,22], respectively.

### 3.2. mRNA Level Expression of SMAD Proteins and Target Genes of TGF-β Pathway in Stable and Progressive U-CLL Cases

**Figure 2:**
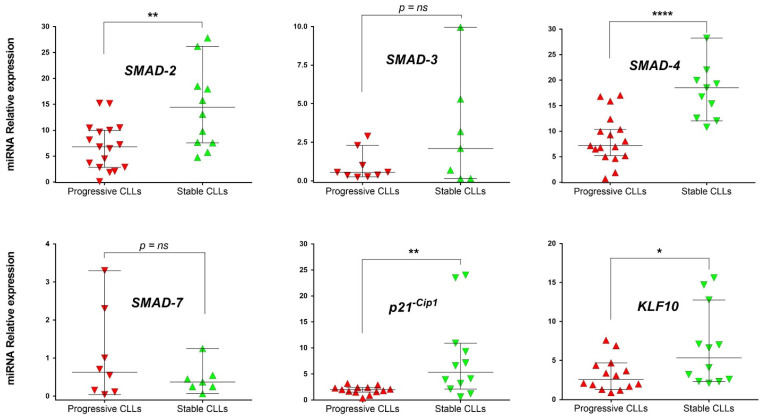
mRNA levels expression of SMAD proteins and of specific target genes modulated by TGF-β pathway activation. Relative expression of SMAD-2, SMAD-3, SMAD-4, SMAD-7, p21^−Cip1^, and KLF10 mRNAs were compared by q-PCR between progressive and stable U-CLL subgroups. Mann–Whitney Unpaired test and median with 95% CI are depicted. GAPDH was used as an endogenous control, and cDNA of MEC-1 cells were used as a reference sample. The relative expression was calculated as 2^−ΔΔCt^. In all cases, *p* < 0.05 was considered statistically significant, (* = *p* < 0.05, ** = *p* < 0.01, and **** = *p* < 0.0001, ns = not significant).

TGF-β protein is a pleiotropic cytokine and exerts its effect on gene expression through transcription factors known as SMAD proteins. Specifically, in CLL, different works support the role of TGF-β pathway as a growth inhibitor of CLL cells [40], and recently, SMAD protein expression has been correlated with disease progression [29]. In view of these data and our previous results, we evaluated the activation of the TGF-β axis in stable and progressive U-CLLs by comparing mRNA levels of SMAD-2, SMAD-3, SMAD-4, and SMAD-7 as well as of two recognized target genes of the TGF-β pathway, cyclin-dependent kinase inhibitor 1A (p21^−Cip1^) [41] and KLF10 (Kruppel Like Factor 10) [42]. Our results showed that SMAD-2 and SMAD-4 mRNAs were significantly decreased in the progressive U-CLL subgroup (*p* = 0.006, *n* = 28 and *p* ≤ 0.0001, *n* = 27, respectively, Mann–Whitney Unpaired test), whereas SMAD-3 and SMAD-7 were not significantly changed (Figure 2). Interestingly, in progressive U-CLL, we also found a significant down-regulation of p21^−Cip1^ and KLF10 mRNA expression compared with the stable cases (*p* = 0.006, *n* = 25 and *p* = 0.032, *n* = 26, respectively, Mann–Whitney Unpaired test), Figure 2. Altogether, these results showed that in the progressive U-CLL subgroup key genes of the TGF-β/SMAD pathway (SMAD-2, SMAD-4, p21^−Cip1^ and KLF10) are decreased, suggesting that the signaling provided for this axis could be inhibited in the CLL cells.

### 3.3. Up-Regulation of miR-26b-5p in Progressive U-CLL Is Associated with Decreased Expression and Nuclear Localization of SMAD-4, Down-Modulation of p21^−Cip1^ Cell Cycle Marker, and Up-Regulation of c-Myc Oncogene

**Figure 3:**
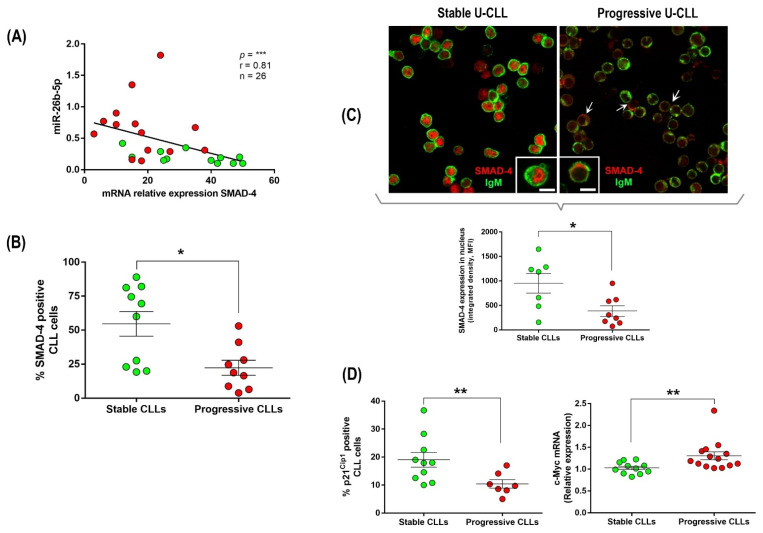
(**A**–**C**) Evaluation of mRNA and protein expression levels and cellular localization of SMAD-4 in CLL cells. (**A**) Correlation between miR-26b-5p and SMAD-4 mRNA expression in the U-CLL subgroups. Red circles = Progressive U-CLL, and green circles = Stable U-CLL (*p ≤* 0.0001, Spearman’s rank test, *r =* 0.81, *n* = 26). (**B**) SMAD-4 protein expression evaluated by flow cytometry in CLL cells (CD19+/CD5+) in the different U-CLL subgroups (*p* = 0.013, Mann–Whitney Unpaired test, *n* = 19). (**C**) Confocal microscopy of Smad-4 (red) and IgM (green) from two representative patients; stable (CLL#10) and progressive (CLL#05) are depicted. Scale bar: 5 µm. Specific fluorescence intensity in nucleus and cytoplasm was measured in the different subgroups. Between 100 and 200 cells were counted in each sample (*n* = 15; stable U-CLLs, *n* = 7; and progressive U-CLLs, *n* = 8). Next, MFI in the nucleus and cytoplasm was used to obtain integrated density and compare mean fluorescence intensities of Smad-4 in the nuclei of CLL samples, (*p* = 0.040, Mann–Whitney Unpaired test, *n* = 15). (**D**) p21^−Cip1^ protein expression evaluated by flow cytometry gating CD19+/CD5+ cells and c-MYC mRNA relative expression from stable and progressive U-CLLs (*p* = 0.007, *n* = 17 and *p* = 0.004, *n* = 25, respectively, Mann–Whitney Unpaired test) are depicted. In all cases, *p* < 0.05 was considered statistically significant, (* = *p* < 0.05, ** = *p* < 0.01, and *** = *p* < 0.001, ns = not significant).

Among the three miRNAs up-regulated in the progressive U-CLL subgroup and potentially involved in TGF-β/SMAD inhibition in other tumors [21,22], it has been previously demonstrated that miR-26b binds the 3′-UTR of the SMAD-4 gene and down-regulates its expression [34]. We evaluated the correlation between miR-26b-5p and SMAD-4 mRNA expression in progressive and stable U-CLL subgroups. Our results show a significant and negative correlation between miR-26-5p and SMAD-4 expression in the progressive subgroup (Figure 3A, *p* ≤ 0.0001, Spearman’s rank test, *r =* 0.81, *n* = 26). This was also confirmed at the protein level by flow cytometry in the same patient cohort (Figure 3B, *p* = 0.013, Mann–Whitney Unpaired test, *n* = 19).

The cytokine TGF-β is a dimer that signals by bringing together two pairs of receptor serine/threonine kinases known as the type I and type II receptors. Phosphorylation and activation of the type I receptors propagate the signal by phosphorylating SMAD transcription factors. Once activated, the receptor substrate SMADs (RSmads) forms a complex with SMAD-4, a binding partner common to all RSmads, and shuttles to the nucleus originating transcriptional activation and repression complexes to control the expression of hundreds of target genes in a given cell [33]. Since SMAD-4 is a key player of TGF-β activation, we performed confocal microscopy with specific antibodies anti-SMAD-4 in order to compare the localization pattern between progressive and stable U-CLLs. Microscopy analysis shows that SMAD-4 expression is higher in stable patients and is mainly localized at the nucleus of leukemic cells, (Figure 3C, left panel). In contrast, progressive cases depict lower levels of SMAD-4 expression, and the protein is mainly visualized at the cytoplasm (white arrows in Figure 3C, right panel). Representative images of leukemic cells expressing SMAD-4 at the different compartments in each different CLL subgroup and an extended microscopy analysis of SMAD-4 localization in seven stable and eight progressive U-CLLs from the validation cohort are shown in Figure 3C (*p* = 0.040, Mann–Whitney Unpaired test, *n* = 15).

TGF-β inhibits progression of cell cycle phase G1 through two sets of events: mobilization of cyclin-dependent kinase (CDK) inhibitors and suppression of the c-Myc oncogene [25]. Considering our previous results, we speculated that tumor cells of progressive U-CLL cases over-expressing miR-26b-5p take advantage of TGF-β inhibition, avoiding the cell cycle arrest that normally occurs in most CLL cells. In order to support this hypothesis, we compared the expression levels of the cyclin-dependent kinase inhibitor p21^−Cip1^ and the transcription factor c-Myc among stable and progressive U-CLLs. Our results show that progressive cases have lower expression levels of p21^−Cip1^ protein and higher expression of the c-Myc gene compared with stable U-CLLs, Figure 3D (*p* = 0.007, *n* = 17 and *p* = 0.004, *n* = 25, respectively, Mann–Whitney Unpaired test). These results suggest not only that TGF-β/SMAD axis is blocked after SMAD-4 inhibition by miR-26b-5p but also that the function of this pathway as a cell cycle regulator is affected in the CLL cells of these progressive and unmutated patients.

### 3.4. Inhibition of miR-26b-5p Recovers TGF-β/SMAD Function Regulating Cell Cycle Progression Molecules in Primary CLL Cells of Progressive Unmutated Patients

**Figure 4:**
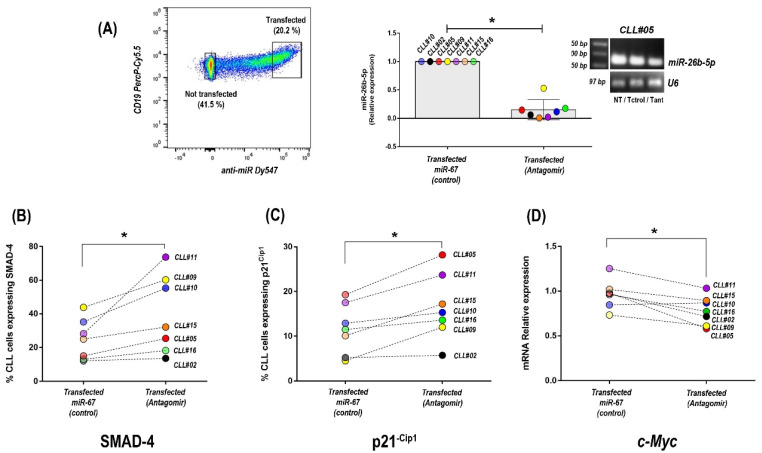
Inhibition of miR-26b-5p and evaluation of SMAD-4, p21^−Cip1^, and c-MYC expression changes in primary CLL cells of progressive U-CLL patients. (**A**) Representative dot plots showing transfected CLL cells with specific antagomir targeting miR-26b-5p labeled with Dy547 (left panel). Graph shows relative expression levels of mir-26b-5p in CLL cells after transfection with the specific antagomir and the irrelevant miR-67 control (*p* = 0.015, signed-rank test, *n* = 7). Agarose gel 5% stained with Ethidium Bromide shows a representative patient after transfection with the specific antagomir (Tant) and the corresponding controls (not transfected—NT—and transfected with the irrelevant miR-67, Tctrol) (right panel). (**B**–**D**) Percentages of cells expressing Smad-4 (**B**), p21^−Cip1^ (**C**), and mRNA levels expression of c-MYC (**D**) after transfection with the specific inhibitor of miR-26b-5p and with miR-67 (control) in each patient (*p* = 0.031, *p* = 0.015, and *p* = 0.047, respectively, Wilcoxon signed-rank test, *n* = 7). Each color corresponds to a single patient. In all cases, *p* < 0.05 was considered statistically significant, (* = *p* < 0.05).

Our results suggest that tumor cells of progressive U-CLL acquire the capacity to progress through the G1 phase of the cell cycle following the impairment of TGF-β/SMAD signaling. This inhibition probably initiates with the up-regulation of miR-26b-5p and the decreased expression of SMAD-4 and is followed by the down-modulation of p21^−Cip1^ and up-regulation of c-Myc genes, which finally allows for restarting of the cell cycle in CLL cells. To confirm this hypothesis, we inhibited miR-26b-5p by transfection with a specific antagomir in progressive U-CLL, and we evaluated SMAD-4, p21^−Cip1^, and c-Myc expression. As previously described [33], transfection experiments with labeled antagomirs showed more than 40% of transfected CLL cells (Figure 4A). After sorting transfected and nontransfected cells by FACS, leukemic cells were isolated to obtain mRNAs and miRNAs, whereas protein expression was visualized by flow cytometry in the corresponding subsets. Quantitative PCR analysis showed that CLL cells incorporating the specific antagomir of miR-26b-5p have decreased values of miR-26b-5p, compared to cells that were transfected with the irrelevant antagomir (miR67, control) (*p* = 0.031, Wilcoxon signed-rank test, *n* = 7) (Figure 4A, middle panel). A representative patient showing this inhibition with the specific antagomir for mir-26b-5p and the corresponding controls (not transfected—NT—and transfected with the irrelevant miR-67, -T ctrol-) are depicted in agarose gel.

In agreement with previous results highlighting the role of miR-26b-5p on SMAD-4 expression [34], our results show that inhibition of miR-26b-5p in primary CLL cells significantly increases the percentage of cells expressing SMAD-4 (Figure 4B), (*p* = 0.031, Wilcoxon signed-rank test, *n* = 7). Furthermore, supporting the hypothesis about the existence of an impairment of TGF-β/SMAD signaling in progressive U-CLL, our results show that after inhibition of miR-26b-5p, two target genes of this pathway (p21^−Cip1^ and c-Myc), both involved in regulation of cell cycle progression at G1 and S phase [35], are affected. Transfection of primary CLL cells from U-cases with the specific antagomir targeting miR-26b-5p results in up-regulation of the cyclin-dependent kinase inhibitor p21^−Cip1^ and down-modulation of the oncogene c-Myc (*p* = 0.015 and *p* = 0.031, respectively, Wilcoxon signed-rank test, *n* = 7, Figure 4C,D). Altogether, these results show that inhibition of miR-26b-5p in progressive U-CLL unlocks the TGF-β/SMAD pathway, which in turn affects the expression of specific target genes (p21^−Cip1^ and c-Myc), both of which are required to inhibit cell cycle progression through the G1 phase.

## 4. Discussion

CLL is the prototype of a cancer where both microenvironmental factors and genetic changes in tumor cells promote the onset, expansion, and progression of the disease [2]. Molecular and clinical heterogeneity are hallmarks of CLL [43]. This leukemia develops through accumulation of malignant B cells that circulate in the PB and are continuously supported by microenvironment signals within BM and secondary lymphoid organs. Even though available treatments often induce remissions, most patients eventually relapse; thus, CLL remains an incurable disease [1]. The basis of this refractoriness is mainly related to the prominent clinical and biologic heterogeneity of CLL cells. A first layer of this heterogeneity is represented by the IgHV mutational status, which separates CLL patients into two different prognostic subgroups, mutated and unmutated cases [5,6]. A second layer underlines the importance of the microenvironment, which continuously boosts different behaviors during leukemia evolution [44]. An additional layer of this heterogeneity exists within the tumor clone itself [32,45], in which a dynamic process exists, leading to an accumulation of the malignant clone, reflecting a balance between cell proliferation and death in the same patient [46]. A better understanding of these different heterogeneity layers and their roles in tumor evolution will help to improve the efficacy of CLL therapy.

Considerable research efforts have identified the molecular pathways in leukemic cells that contribute to antiapoptotic and survival signaling. Some of them such as BCR and NF-kB [47,48], Pi3K/AKT [33,49], WNT [50], NOTCH-1 [51], and IL-4/CD40L [38], are boosted after microenvironment interactions during disease progression [52]. Different works illustrate how miRNAs are often involved in the regulation of these pathways in leukemias in general [53] and in CLL in particular [54]. miRNAs can function both as oncogenes and tumor suppressors, depending on the target gene, and the mechanism can be related to the different cancer hallmarks [8]. In CLL, miRNAs have been implicated at most levels, both in the pathogenesis and evolution of the disease [11,12,13,14,55], and different miRNAs signatures correlating with clinical outcomes have been proposed [12,14,37,56]. Furthermore, miRNAs have been involved in therapy refractoriness of CLL [16] and also linked with cell proliferation and disease progression [33].

miRNAs signatures in CLL and their association with M-CLL and U-CLL profiles [12,56], therapy resistance [16], and/or activated CLL cells [17] have been previously performed by different groups. Specifically in this work, we focused on those patients with U-IgHV profile and different clinical outcomes comparing the miRNome of stable and progressive U-CLLs. We concentrated on those miRNAs with different relative abundance identified in the progressive U-CLL subgroup, identifying 21 differentially expressed miRNAs (3 down-regulated and 18 up-regulated). Of these, 7 are reported here for the first time (Figure 1B, red names), while 13 were previously linked to CLL evolution (Figure 1B, black labels). All of them depict a similar up-/down-modulation profile according with the IgHV status, or the previously described activation profile of CLL cells (see references in Figure 1B), except for Let-7i-5p, which was only found in M-CLL [36]. The consistency of our results with these previous reports supports the technical reliability of our initial screening approach.

Next, we validated the miRNome results by q-PCR in an extended CLL cohort of 15 patients focusing on the miRNAs that kept statistically significant differences between stable and progressive U-CLLs. Interestingly, all the three selected miRNAs displaying this pattern (miR-26b-5p, miR-106b-5p, and miR-142-5p) were previously described as targeting genes involved in the TGF-β/SMAD signaling [21,22,57], a key pathway in animal cells whose misregulation can result in tumor development [25]. Specifically in CLL, the TGF-β pathway has been described as an axis that can contribute to the clinical and biological progression, albeit not in all patients [27,30], which underlines the importance of the different heterogeneity layers that exist in CLL. In normal and premalignant cells, TGF-β can enforce homeostasis and restrain tumor progression directly through modulation of oncogenes and/or tumor-suppressors gene or indirectly through microenvironment signaling [23,25]. However, when cancer cells loose TGF-β tumor-suppressive responses, they can use TGF-β to their advantage to initiate immune evasion, growth factor production, differentiation into an invasive phenotype, and metastatic dissemination or to establish and expand metastatic colonies.

The cytokine TGF-β is a dimer that signals by bringing together two pairs of receptor serine/threonine kinases known as the type I and type II receptors. Phosphorylation and activation of the type I receptors propagate the signal by phosphorylating SMAD transcription factors. Once activated, the receptor substrate SMADs (RSmads) forms a complex with SMAD-4, a binding partner common to all RSmads, and shuttles to the nucleus originating transcriptional activation and repression complexes to control the expression of hundreds of target genes in a given cell [25]. In CLL, an interesting work of Witkowska et al. recently linked the expression levels of SMADs proteins with clinical evolution and shows that lower SMAD-4 protein levels correlate with progressive course of the disease [29]. From the three miRNAs up-regulated in the U-CLL subgroup, two of them, miR-26b-5p and miR-142-5p, have been described as targeting different SMADs proteins. Specifically, miR-26b-5p targets SMAD-4 [34], and miR-142-5p targets SMAD-3 [22]. Among these, the miR-26b is the only miRNA that has been demonstrated to bind the 3′-UTR of the SMAD-4 gene and down-regulate its expression [34]. Considering the relevance of SMAD-4 as a binding partner common to all RSmads, indispensable for shuttling SMAD-4-RSmads complexes to the nucleus and to activate or repress hundreds of target genes at once, we decided to evaluate the expression levels and cellular localization of SMAD-4 in our CLL cohort. In agreement with the results of Witkowska et al., SMAD-4 expression at both mRNA and protein levels remained lower in progressive CLL cases compared with the stable disease. Interestingly, when 26 U-CLLs were interrogated regarding SMAD-4 mRNA expression and miR-26b-5p, a negative significant correlation was found, suggesting that this miRNA could be involved in the down-modulation of SMAD-4 and, in consequence, could impair the TGF-β/SMAD pathway. Nuclear localization of SMAD-4 is a hallmark of the canonical TGF-β activation. Remarkably, our microscopy results are in agreement with our previous observations, demonstrating that, in the cases of a more aggressive disease, SMAD-4 is not only down-modulated but also excluded from the nucleus.

Altogether, our results suggest the existence of a TGF-β inactivation in primary CLL cells of progressive U-cases compared to stable patients. Although our data are not direct evidence of the cause of disease progression, they are in agreement with previous reports linking impairment of TGF-β pathway with poor clinical outcome in CLL [27,28,29,30]. In addition, these works propose that TGFβ-1 induces growth arrest in CLL cells, but about one-third of the patients are resistant to its effects [26,58]. Interestingly, results from D’Abundo et al. [14] postulate an antileukemic activity of miR-26 in CLL. Indeed, despite different studies regarding the role of TGF-β/SMAD pathway on CLL clinical progression [28,29], the molecular mechanisms responsible for TGF-β signaling in CLL, as well as the heterogeneity of the functional activity of this pathway among CLL patients with different clinical outcomes, are not completely elucidated yet.

Two well-characterized outcomes of the TGF-β pathway activation are growth-arrest and apoptosis [39]. TGF-β inhibits cell cycle progression through expression of p21^−Cip1^, which inhibits cyclinE/A-cdk2 complexes and down-regulation of c-Myc oncogene [25]. Indeed, TGF-β is frequently present in the tumor microenvironment, initially as a signal to prevent premalignant progression, but eventually as a factor that malignant cells may use to their own advantage [25,39]. In the case of progressive CLL patients, inhibition of the TGF-β pathway could be used to unblock the classical G0/G1 arrest in leukemic cells, and this action could be orchestrated by miR-26b-5p expression. In order to investigate this hypothesis, we compared the expression levels of p21^−Cip1^ inhibitor and c-Myc oncogene in the progressive and stable patients of our cohort as well as after and before inactivation of miR26b-5p by specific antagomir molecules. Our results showed that specific inhibition of the miR-26b-5p significantly increases the expression of p21^−Cip1^ protein, while it down-regulates c-Myc.

In summary, we provide for the first time evidence about miR-26b-5p affecting tumor progression in U-CLL through inactivation of TGF-β/SMAD pathway. Furthermore, we highlight miR-26b-5p as a novel key molecule in CLL, thus identifying another piece of the complex puzzle of clinical aggressiveness in this leukemia. Our results highlight the possibility that suppression of TGF-β signaling by miR-26-5p could be beneficial for the “fitness” of the leukemic clone and suggest that TGF-β pathway modulation could become an alternative strategy to explore in CLL therapy.

## 5. Conclusions

This work focused on a subgroup of CLL patients with the poorest clinical outcome. We describe a new molecular mechanism in the progressive U-CLL subgroup, linking for the first time the TGF-β/SMAD pathway with miR-26b-5p and regulation of key molecules involved in the cell cycle regulation of CLL. Overexpression of miR-26b-5p down-modulates the TGF-β/SMAD pathway in primary CLL cells of these patients. Our results postulate a key role for the miR-26b-5p in CLL cells and underline the importance of the TGF-β molecule as an essential immunoregulatory molecule in CLL. Although TGF-β is frequently present in the tumor microenvironment, initially as a signal to prevent premalignant progression, our data suggest that malignant cells can circumvent this suppressive effects by reactivation of the cell cycle arrest in CLL. Our work highlights the relevance of microenvironment interactions and genetic modifications that exist in the leukemic clone during disease evolution.

## Figures and Tables

**Table 1:** Clinical and molecular characterization of CLL patients, AID and LPL expression assessed by q-PCR as described in [34,35]. T.F.T = Time from initial diagnosis to first treatment for clinical progression. L.C. = Lymphocyte Count. N/D = no determinate; N/T = not treated: Refers to patients who did not receive any treatment at 4 years follow-up.

Rational Study	CLL #	Age	Sex	Stage Binet	L.C. (×10^3^/µL)	FISH	AID	LPL	IgHV Status Homology (%)	IgHV Rearrangement	Clinical Disease Status	T.F.T. (Years)
Study cohort (progressive patients)	1	58	M	C	62.000	del (17p)	(+)	(+)	100	VH1-02	Progressive	<1
	2	52	M	B	26.200	del (13q)	(+)	(+)	98.0	VH4b*02	Progressive	2.4
	3	66	F	B	59.250	Normal	(+)	(+)	99.6	VH2-5*10	Progressive	3
	4	58	M	C	72.100	del (13q)/del (11)	(+)	(+)	100	VH3-48*03	Progressive	<1
	5	72	M	B	56.200	Normal	(+)	(+)	100	VH3-11*01	Progressive	<1
	6	53	M	B	116.000	Normal	(+)	(+)	99.0	VH1-69	Progressive	<1
Study cohort (stable patients)	15	66	F	A	7520	Normal	(−)	(+)	100	VH3-9*01	Stable	N/T
	16	67	M	A	9520	Normal	(−)	(+)	100	VH3-13*01	Stable	N/T
	17	72	M	A	10.830	del (13q)	(−)	(+)	99.7	VH3-23*01	Stable	N/T
	18	64	M	B	15.960	Normal	(−)	(+)	100	VH1-69*01	Stable	N/T
	19	63	F	A		Normal	(−)	(+)	100	VH2-5*01	Stable	N/T
Validation cohort (progressive patients)	7	59	M	B	26.200	Normal	(+)	(+)	100	VH3-7*01	Progressive	1
	8	77	M	B	34.600	Normal	(+)	(+)	99.1	VH1-69*12	Progressive	1.7
	9	55	M		300.250	del (17p)	(+)	(+)	99.2	VH3-15*01	Progressive	2.8
	10	53	F	C	55.360	del (17p)	(+)	(+)	100	VH1-69	Progressive	<1
	11	49	M	B	154.830	del (13p)	(+)	(+)	100	VH1-69*01	Progressive	1.5
	12	59	M	C	94.000	Normal	(+)	(+)	100	VH4-34*01	Progressive	1.2
	13	64	M	B	64.740	Normal	(+)	(+)	100	VH1-2*02	Progressive	2.5
	14	52	M	C	421.000	Tris12	(+)	(+)	100	VH1-69*01	Progressive	<1
Validation cohort (stable patients)	20	67	M	A	9.179	Normal	(−)	(+)	100	VH1-69*13	Stable	N/T
	21	61	M	A	6.230	Normal	(−)	(+)	98.6	VH3-30	Stable	N/T
	22	79	M	A	8.900	Normal	(−)	(+)	100	VH1-69*01	Stable	N/T
	23	73	M	A	18.800	Normal	(−)	(−)	100	VH4-30-2*01	Stable	N/T
	24	67	F	A	19.660	del (13q)	(−)	(+)	100	VH2-5*01	Stable	N/T
	25	73	F	A	N/D	N/D	(−)	(+)	98.2	VH1-18*01	Stable	N/T
	26	72	M	A	10.830	del (13q)	(−)	(−)	99.7	VH3-23*01	Stable	N/T

**Table 2:** List of the most differentially expressed miRNAs between progressive and indolent patients, *p* ≤ 0.05 in Volcano analysis. Fold changes, *p*-values, and the main bibliographical data reporting miRNAs previously described in CLL are provided.

miRNAs	Fold Change	*p* Value	Previously Reported in CLL	References
**Increased relative expression in progressive U-CLL**				
Let-7g-5p	1.70	0.005	YES	[19]
Let-7i-5p	1.79	0.007	YES	[36]
miR-7-5p	2.49	0.005	YES	[13,14]
miR-19a-3p	3.22	<0.0001	YES	[11,37]
miR-19b-3p	2.74	<0.0001	YES	[11]
miR-142-3p	2.54	<0.0001	YES	[17]
miR-26a-2-3p	7.64	0.01	YES	[14]
miR-29b-3p	2.30	0.01	YES	[38]
miR-29c-5p	5.68	0.002	YES	[38]
miR-106b-5p	2.55	0.005	YES	[14]
miR-142-5p	1.76	0.03	YES	[22]
miR-374b-5p	1.60	0.004	YES	[39]
**miR-7-1-3p**	3.31	0.008	NO	--
**miR-26b-5p**	3.33	0.02	NO	--
**miR-140-5p**	2.51	0.02	NO	--
**miR-340-5p**	1.63	0.01	NO	--
**miR-374a-3p**	2.60	0.02	NO	--
**miR-3613-5p**	3.63	0.006	NO	--
**Reduced relative expression in progressive U-CLL**				
miR-146a-5p	0.56	0.01	YES	[12,17]
**miR-130b-5p**	0.40	0.01	NO	--
**miR-505-5p**	0.42	0.008	NO	--

## Data Availability

The sequence data are deposited in DRYAD: doi:10.5061/dryad.n2z34tmzd. Data are accessible for peer review at the following link: https://datadryad.org/stash/share/flVsy1kgg770p5Ef6vAoYLRL1SpafGnt2oricQ8X6oo, accessed on 10 February 2022.

## Supplementary Materials

The following supporting information can be downloaded at: https://www.mdpi.com/article/10.3390/cancers14071676/s1, Table S1: miRNAs quantification; Table S2: Primer sequences.
