# Supplementary material for: TGF-β/SMAD Pathway Is Modulated by miR-26b-5p: Another Piece in the Puzzle of Chronic Lymphocytic Leukemia Progression
Source: Cancers (Basel). 2022 Mar 25;14(7):1676. doi: 10.3390/cancers14071676 (PMC8997107; doi:10.3390/cancers14071676)
Supplement: Supplementary file 1 [file cancers-14-01676-s001.zip › Supplementary Table S1, miRNAs quantification.pdf]

Table S1. miRNAs quantification

**miRNA Copy number**				
	**Progressive U-CLL**	**Stable U-CLL**	**Ratio**	**p-value**
hsa-miR-320d	0	3,368248698	0	5,34967E-07
hsa-miR-199a-3p	49,91845618	165,114832	0,302325694	0,000385909
hsa-miR-125a-5p	695,614177	2663,107868	0,261203906	0,002290477
hsa-miR-584-5p	0	42,32120514	0	0,002624039
hsa-miR-19a-3p	126,3814747	39,38668568	3,20873596	0,004777564
hsa-miR-7-5p	566,4835211	252,6014073	2,242598437	0,005746643
hsa-miR-34a-5p	96,34024958	13,14085074	7,331355594	0,007615796
hsa-miR-139-5p	0	21,65443511	0	0,007935292
hsa-miR-142-3p	2350,117054	980,2907213	2,397367438	0,009422079
hsa-let-7g-5p	66201,66043	38117,29019	1,736788216	0,014332678
hsa-miR-942	0,831998669	7,434188459	0,111915197	0,015519724
hsa-miR-3912	3,671550825	0	-	0,017241755
hsa-miR-769-5p	203,2663465	340,5723598	0,596837473	0,017686705
hsa-miR-409-5p	0	26,70803995	0	0,017960356
hsa-miR-146a-5p	10771,99971	23256,82483	0,463175855	0,018171124
hsa-miR-19b-3p	731,0875412	269,0524679	2,717267553	0,018830696
hsa-miR-133a	0	5,484870489	0	0,018945282
hsa-miR-2110	11,90080054	23,31377906	0,510462097	0,020597268
hsa-miR-425-5p	587,1195757	813,653997	0,72158384	0,020781443
hsa-miR-4424	3,12895891	0	-	0,02240262
hsa-miR-454-5p	0,693332224	11,66743204	0,059424578	0,023241231
hsa-miR-106b-5p	263,2833054	115,3211083	2,283045223	0,026510391
hsa-miR-493-3p	0	2,887422254	0	0,026890407
hsa-miR-320c	4,328661456	16,19311323	0,267314963	0,028785533
hsa-miR-126-3p	90,50857503	711,0074054	0,127296248	0,028899352
hsa-miR-330-3p	24,02441858	88,20151141	0,272381031	0,029106853
hsa-miR-7-1-3p	100,9338041	30,23620171	3,338177362	0,029173977
hsa-miR-1307-3p	56,60408122	107,3006683	0,52752776	0,034038907
hsa-miR-3609	5,305180509	48,54593673	0,109281659	0,03722726
hsa-miR-1468	51,85356649	31,27835476	1,65780991	0,037728093
hsa-miR-598	64,44769925	20,13145562	3,201343235	0,038341607
hsa-miR-29c-5p	28,70907604	4,928471056	5,825148553	0,044007965
hsa-miR-181c-3p	0,693332224	4,546766205	0,152489086	0,045963492
hsa-miR-374b-5p	769,2182516	465,9226412	1,650957012	0,0468327
hsa-miR-199a-5p	70,78318222	1402,082397	0,050484324	0,047099112
hsa-miR-15a-5p	413,8523788	196,9572661	2,101229303	0,052114711
hsa-miR-374b-3p	15,81346266	4,810728032	3,287124642	0,053389961
hsa-miR-1343	0	5,414224675	0	0,055164497
hsa-miR-1301	23,54804993	80,64711634	0,291988741	0,057379434
hsa-miR-487b	0	3,224493482	0	0,059052001
hsa-miR-758	0	6,448986965	0	0,059052001

hsa-miR-130a-3p	0	17,1572297	0	0,060298528
hsa-miR-493-5p	1,591554153	7,988124305	0,199240033	0,068802203
hsa-miR-409-3p	0	24,73763696	0	0,07061368
hsa-miR-340-3p	81,64160643	141,1926292	0,57822853	0,070676455
hsa-miR-191-3p	4,503549493	11,66743204	0,385993206	0,072408073
hsa-let-7i-5p	31251,30105	17013,45332	1,836858188	0,072664144
hsa-miR-142-5p	12410,71072	8237,4186	1,506626204	0,073123908
hsa-miR-16-1-3p	2,990292465	0,601033056	4,975254584	0,074120139
hsa-miR-3613-5p	164,1452132	45,81596395	3,582707839	0,075266761
hsa-miR-370	0	32,77532207	0	0,077477036
hsa-miR-335-5p	2834,928964	1047,713156	2,70582549	0,077803101
hsa-miR-664-5p	79,82064886	217,0374955	0,367773544	0,078650263
hsa-miR-323b-3p	0	3,104286871	0	0,080828725
hsa-miR-146b-3p	26,67737483	75,65430296	0,352622042	0,082863918
hsa-miR-340-5p	536,8410995	356,2315349	1,507000495	0,084622271
hsa-miR-26a-2-3p	11,19539445	1,562685945	7,164199876	0,08641557
hsa-miR-505-5p	31,9046242	80,51075188	0,396277807	0,087886658
hsa-miR-33b-3p	2,839552156	3,945733149	0,719651342	0,090524542
hsa-miR-127-3p	2,839552156	114,811517	0,024732294	0,090539506
hsa-miR-326	1,856813178	35,79171801	0,051878291	0,091053173
hsa-miR-29b-3p	3807,226975	1572,491454	2,421143191	0,091699986
hsa-miR-15b-5p	2061,703659	1389,00081	1,484307024	0,092282002
hsa-miR-140-5p	134,2426545	52,60202214	2,552043611	0,092671912
hsa-miR-361-3p	2657,107001	3331,634954	0,797538457	0,097266504
hsa-miR-4326	0,831998669	4,089488365	0,203448108	0,097539584
hsa-miR-130b-5p	74,69584434	213,1884203	0,350374773	0,100665314
hsa-let-7e-5p	22,72849111	66,63004005	0,341114775	0,100777572
hsa-miR-99b-5p	87,59731166	368,3477668	0,237811437	0,102405214
hsa-miR-4524a-3p	13,05842755	5,291554476	2,467786661	0,103502765
hsa-miR-29b-1-5p	9,037100654	2,887422254	3,129816099	0,104286001
hsa-miR-744-5p	74,25533129	219,6771138	0,338020334	0,106928736
hsa-miR-500a-5p	6,221696227	1,562685945	3,981411779	0,111589144
hsa-miR-374a-3p	78,47130396	29,58807145	2,652126351	0,112640842
hsa-miR-624-5p	4,479401765	3,608661921	1,24129161	0,121369765
hsa-miR-1273c	0,970665114	5,508419094	0,176214826	0,122795477
hsa-miR-641	9,983618039	4,306352983	2,318346424	0,126158637
hsa-miR-101-3p	5632,338615	2018,696371	2,790087056	0,127910047
hsa-miR-20a-5p	1096,177259	401,1438848	2,732628617	0,131630857
hsa-miR-93-3p	7,270658464	14,00338203	0,519207321	0,132438482
hsa-miR-889	0	10,54093892	0	0,133314504
hsa-miR-590-3p	667,5150159	118,206067	5,647045308	0,138815259
hsa-miR-454-3p	25,93574714	14,69860951	1,764503447	0,139319106
hsa-miR-26b-5p	20272,80686	5950,562499	3,406872352	0,139956645

hsa-miR-296-3p	0	3,321151489	0	0,140704441
hsa-miR-126-5p	31,69900252	378,721622	0,083700007	0,141191519
hsa-miR-146b-5p	5075,277811	10398,52139	0,488076874	0,149281707
hsa-miR-181d	6,553178512	15,7829326	0,415206646	0,151855485
hsa-miR-654-3p	0	47,94106376	0	0,155473353
hsa-miR-4485	8,729766097	586,0293325	0,014896466	0,156323146
hsa-miR-20b-5p	41,6954264	15,92176064	2,61876983	0,159799718
hsa-miR-450b-5p	1,109331558	8,706900385	0,127408321	0,161736367
hsa-let-7i-3p	69,26480322	38,45981206	1,800965723	0,162482436
hsa-miR-193a-5p	5,034067544	6,736497397	0,747282638	0,163934553
hsa-miR-550a-3p	4,756734655	2,406595809	1,976540737	0,164264851
hsa-miR-548j	1,386664448	4,546766205	0,304978173	0,165376019
hsa-miR-651	15,97042289	3,365785111	4,74493242	0,165572089
hsa-miR-21-5p	28241,26304	15055,12872	1,875856631	0,166562948
hsa-miR-5010-3p	0	2,286389198	0	0,169496121
hsa-miR-30e-5p	19530,16537	11312,69978	1,726392969	0,171495002
hsa-miR-224-5p	0	43,99533061	0	0,17606662
hsa-miR-3681-5p	5,208955582	10,44181732	0,498855268	0,179658703
hsa-miR-652-3p	318,332786	547,6185032	0,581303926	0,180555547
hsa-miR-106b-3p	276,0936697	360,7930827	0,765241029	0,184164254
hsa-miR-28-3p	3932,176776	5077,74497	0,774394303	0,184587048
hsa-miR-185-5p	657,3228465	860,7434711	0,763668699	0,186411103
hsa-miR-29a-5p	7,270658464	1,682892556	4,320334319	0,187289063
hsa-miR-4772-5p	3,345922478	8,419389953	0,39740676	0,188100654
hsa-miR-1255a	1,802663782	5,341115273	0,337506998	0,188762325
hsa-miR-4524b-3p	24,60359808	8,053842945	3,054889231	0,192353733
hsa-miR-155-3p	5,263104977	1,562685945	3,36798638	0,192709665
hsa-miR-29a-3p	32814,57348	17744,44401	1,849287216	0,192921815
hsa-miR-194-5p	40,04350292	19,96415179	2,005770309	0,194944816
hsa-miR-421	16,2473898	39,47348934	0,411602574	0,195099719
hsa-miR-16-5p	21533,2925	17436,84724	1,234930387	0,195473195
hsa-miR-150-5p	118770,5198	84939,38025	1,398297462	0,205569897
hsa-miR-379-5p	0	2,623460427	0	0,206223534
hsa-miR-495	0	2,623460427	0	0,206223534
hsa-miR-29c-3p	981,3640102	357,7501654	2,743154595	0,208623696
hsa-miR-20a-3p	7,240656797	2,767215643	2,61658567	0,220119106
hsa-miR-9-5p	38,97661288	12,05160048	3,234144125	0,221064528
hsa-miR-98	608,8697178	838,5063353	0,726136097	0,226240922
hsa-miR-625-3p	51,53379208	23,49724073	2,193184837	0,226350331
hsa-miR-32-3p	2,978218601	1,0818595	2,752870036	0,227164465
hsa-miR-629-3p	13,30539279	3,005165278	4,427507827	0,231303973
hsa-miR-1226-3p	4,509403432	19,83025093	0,22740022	0,23143373
hsa-miR-671-5p	9,290285815	2,644545445	3,512999118	0,233169338

hsa-miR-2964a-5p	0	2,960531655	0	0,234295037
hsa-miR-660-5p	110,6470411	55,50806583	1,993350687	0,237651056
hsa-miR-22-3p	1894,797558	3035,335209	0,624246559	0,23777894
hsa-miR-328	32,00633708	91,51882299	0,34972409	0,239492789
hsa-miR-155-5p	36532,98913	65569,21578	0,557166785	0,241220613
hsa-miR-191-5p	29793,88216	55012,39717	0,541584874	0,242748944
hsa-miR-1277-3p	7,921915157	3,585113316	2,209669391	0,244202358
hsa-let-7f-2-3p	16,16909267	8,732912576	1,851512028	0,244990114
hsa-miR-423-5p	4220,889083	8820,002196	0,478558734	0,245454676
hsa-miR-644b-5p	1,591554153	4,186146371	0,380195533	0,24589586
hsa-miR-32-5p	122,3616117	56,04830741	2,183145529	0,250732981
hsa-miR-374a-5p	2286,374401	1426,168201	1,603159009	0,250957107
hsa-miR-125b-5p	102,9878231	213,8329998	0,48162736	0,251871542
hsa-miR-3909	2,550145402	9,788759885	0,26051772	0,252390617
hsa-miR-376c	0	3,297602884	0	0,256223112
hsa-miR-361-5p	350,9204967	555,5811244	0,631627824	0,256968657
hsa-miR-181b-5p	229,2408753	624,5050134	0,367076117	0,264782508
hsa-miR-579	3,25555149	1,322272722	2,462087764	0,281744287
hsa-miR-18a-3p	8,367916159	21,8762269	0,382511856	0,28326292
hsa-miR-381	1,247998003	16,03073658	0,077850322	0,288940582
hsa-miR-4677-3p	7,282732328	4,666972816	1,560483126	0,291851622
hsa-miR-484	831,9496322	1242,338821	0,669664039	0,2923773
hsa-miR-329	0	5,103165638	0	0,297989383
hsa-miR-410	0	7,028935003	0	0,302459899
hsa-miR-9-3p	15,37368158	3,705319927	4,149083449	0,302804624
hsa-miR-138-5p	77,62503551	0	-	0,31049176
hsa-miR-210	13,0280599	3,966818167	3,284259412	0,314960496
hsa-miR-339-3p	456,6725027	288,6176697	1,582274928	0,315196118
hsa-let-7g-3p	6,456953585	1,202066111	5,371546144	0,318466795
hsa-miR-342-5p	341,6920441	593,7627023	0,575469026	0,322821919
hsa-miR-138-1-3p	7,198215279	0	-	0,323056829
hsa-miR-122-5p	10,93598936	80,67834476	0,135550492	0,327887185
hsa-miR-27b-5p	13,07013543	0,601033056	21,74611747	0,329675897
hsa-miR-1	1,591554153	5,824405305	0,273256078	0,332923631
hsa-let-7a-3p	109,5721012	46,24476601	2,369394651	0,3342639
hsa-miR-181a-5p	2357,818705	6593,69683	0,357586763	0,334930592
hsa-miR-212-5p	3,231403762	5,989245538	0,539534361	0,335378568
hsa-miR-136-3p	1,326295127	23,78337484	0,05576564	0,339728325
hsa-miR-101-5p	3,611181504	0,721239667	5,006909174	0,34198887
hsa-miR-15b-3p	32,89285113	20,39788103	1,612562162	0,343441134
hsa-miR-26a-5p	260847,9832	117001,9369	2,229433034	0,347293291
hsa-miR-2355-5p	3,508736652	13,99599127	0,25069583	0,348457177
hsa-miR-132-5p	4,202068876	7,408176267	0,567220423	0,355496518

hsa-miR-1295a	94,33233011	76,06448359	1,240162631	0,355944025
hsa-miR-338-5p	10,23094926	19,41021595	0,527090955	0,360218116
hsa-miR-324-3p	22,72849111	10,33776856	2,198587731	0,361845775
hsa-miR-27a-3p	3721,107425	2840,974882	1,309799481	0,367254195
hsa-let-7b-5p	8397,615645	13768,75774	0,609903653	0,373982362
hsa-miR-222-3p	1328,209391	1964,761726	0,676015505	0,377886446
hsa-miR-150-3p	296,2818934	174,651948	1,696413334	0,379071715
hsa-miR-625-5p	9,862879398	20,39788103	0,483524705	0,381183801
hsa-miR-3611	2,158293796	0,961652889	2,244358459	0,383711655
hsa-miR-181a-3p	17,76064683	68,67876867	0,258604619	0,385007363
hsa-miR-181a-2-3p	38,12558845	137,1431363	0,277998517	0,386948915
hsa-miR-501-3p	125,4759349	191,4797863	0,655295983	0,390690103
hsa-miR-450a-5p	14,83657762	31,29204902	0,474132506	0,390785598
hsa-miR-17-5p	428,8598186	263,8611223	1,625324015	0,39435705
hsa-miR-1973	2,218663117	28,2274686	0,078599436	0,396269579
hsa-miR-335-3p	206,7194698	139,6608826	1,480152967	0,397424564
hsa-miR-182-5p	74,95049345	143,8938371	0,520873548	0,398227154
hsa-miR-320a	1234,392943	2128,352133	0,5799759	0,406134913
hsa-miR-2277-5p	3,25555149	6,012794143	0,541437377	0,406379508
hsa-miR-4662a-5p	8,639395109	4,06593976	2,124821227	0,407361916
hsa-miR-22-5p	9,465173853	4,567851223	2,072128314	0,407579461
hsa-miR-190b	3,243477626	1,562685945	2,075578677	0,411284625
hsa-miR-141-3p	127,059807	25,03637807	5,075007521	0,411727695
hsa-miR-451a	105,7263943	2107,597994	0,050164403	0,414436005
hsa-miR-200a-3p	63,82827415	15,61316519	4,088105991	0,416124752
hsa-miR-628-5p	4,238290468	1,803099167	2,350558719	0,417234546
hsa-miR-486-5p	2535,329689	7474,204059	0,33921066	0,418866494
hsa-miR-130b-3p	17,76064683	24,22587115	0,733127272	0,420253005
hsa-miR-378c	10,610727	5,168884278	2,052808001	0,420282214
hsa-miR-548k	22,7467849	30,88679556	0,736456615	0,421805694
hsa-miR-509-3p	1,663997338	0	-	0,422649731
hsa-miR-514a-3p	2,218663117	0	-	0,422649731
hsa-miR-29b-2-5p	1,386664448	0	-	0,422649731
hsa-miR-3614-5p	0	1,202066111	0	0,422649731
hsa-miR-3687	0	2,644545445	0	0,422649731
hsa-miR-4521	0,831998669	0	-	0,422649731
hsa-miR-1273g-3p	0	5,409297501	0	0,422649731
hsa-miR-708-3p	2,634662451	0	-	0,422649731
hsa-miR-378d	1,109331558	0	-	0,422649731
hsa-miR-362-3p	2,634662451	0	-	0,422649731
hsa-miR-548h-3p	3,46666112	0	-	0,422649731
hsa-miR-4524a-5p	1,663997338	0	-	0,422649731
hsa-miR-222-5p	0,693332224	0	-	0,422649731

hsa-miR-548g-5p	0,693332224	0	-	0,422649731
hsa-miR-33a-5p	1,386664448	0	-	0,422649731
hsa-miR-548ar-3p	1,109331558	0	-	0,422649731
hsa-miR-365a-3p	2,773328896	0	-	0,422649731
hsa-miR-30a-3p	1,109331558	0	-	0,422649731
hsa-miR-1285-5p	0,970665114	0	-	0,422649731
hsa-miR-4645-3p	0,831998669	0	-	0,422649731
hsa-miR-4662a-3p	0,831998669	0	-	0,422649731
hsa-miR-944	0,831998669	0	-	0,422649731
hsa-miR-141-5p	0,693332224	0	-	0,422649731
hsa-miR-5000-3p	0,693332224	0	-	0,422649731
hsa-miR-580	0,693332224	0	-	0,422649731
hsa-miR-4772-3p	0	3,726404945	0	0,422649731
hsa-miR-873-5p	0	5,048677667	0	0,422649731
hsa-miR-424-3p	0	2,764752056	0	0,422649731
hsa-miR-4433-3p	0	15,62685945	0	0,422649731
hsa-miR-3690	0	2,524338834	0	0,422649731
hsa-miR-556-5p	0	1,322272722	0	0,422649731
hsa-miR-582-3p	0	0,961652889	0	0,422649731
hsa-miR-371b-5p	0	1,202066111	0	0,422649731
hsa-miR-4423-3p	9,814583941	0	-	0,422649731
hsa-miR-34c-5p	0	0,721239667	0	0,422649731
hsa-miR-1249	0	4,327438	0	0,422649731
hsa-miR-432-5p	0	5,056068428	0	0,422649731
hsa-miR-548a-3p	0	0,721239667	0	0,422649731
hsa-miR-125a-3p	0	0,961652889	0	0,422649731
hsa-miR-550a-3-5p	0	0,601033056	0	0,422649731
hsa-miR-548d-5p	0	1,562685945	0	0,422649731
hsa-miR-656	0	3,033641057	0	0,422649731
hsa-miR-3940-3p	0	2,022427371	0	0,422649731
hsa-miR-3157-3p	0	0,601033056	0	0,422649731
hsa-miR-1976	0	1,202066111	0	0,422649731
hsa-miR-744-3p	0	1,202066111	0	0,422649731
hsa-miR-382-5p	0	0,841446278	0	0,422649731
hsa-miR-1284	0	1,923305778	0	0,422649731
hsa-miR-548s	0	0,961652889	0	0,422649731
hsa-miR-873-3p	0	2,043512389	0	0,422649731
hsa-miR-139-3p	0	4,044854743	0	0,422649731
hsa-miR-1303	1,326295127	0	-	0,422649731
hsa-miR-1254	0	1,442479333	0	0,422649731
hsa-miR-590-5p	0	0,601033056	0	0,422649731
hsa-miR-543	0	0,601033056	0	0,422649731
hsa-miR-4423-5p	1,856813178	0	-	0,422649731

hsa-miR-2355-3p	0	0,601033056	0	0,422649731
hsa-miR-149-5p	0	0,721239667	0	0,422649731
hsa-miR-4685-3p	0	1,322272722	0	0,422649731
hsa-miR-485-3p	0	4,044854743	0	0,422649731
hsa-miR-877-5p	0	2,524338834	0	0,422649731
hsa-miR-1290	14,05872835	0	-	0,422649731
hsa-miR-3917	1,326295127	0	-	0,422649731
hsa-miR-570-3p	0	2,764752056	0	0,422649731
hsa-miR-3130-3p	0	0,961652889	0	0,422649731
hsa-miR-642a-3p	0	0,841446278	0	0,422649731
hsa-miR-26-5p	0	147291,0839	0	0,422649731
hsa-miR-4746-5p	0	1,562685945	0	0,422649731
hsa-miR-3177-3p	0	0,841446278	0	0,422649731
hsa-miR-4781-3p	0	0,841446278	0	0,422649731
hsa-miR-940	0	0,841446278	0	0,422649731
hsa-miR-3157-5p	0	0,841446278	0	0,422649731
hsa-miR-548ab	0	0,841446278	0	0,422649731
hsa-miR-4707-3p	0	0,841446278	0	0,422649731
hsa-miR-615-3p	0	1,322272722	0	0,422649731
hsa-miR-363-5p	0	0,721239667	0	0,422649731
hsa-miR-491-5p	0	0,721239667	0	0,422649731
hsa-miR-1180	0	0,721239667	0	0,422649731
hsa-miR-433	0	1,322272722	0	0,422649731
hsa-miR-3676-3p	0	0,601033056	0	0,422649731
hsa-miR-4433-5p	0	0,601033056	0	0,422649731
hsa-miR-548ap-5p	0	0,601033056	0	0,422649731
hsa-miR-5683	0	0,601033056	0	0,422649731
hsa-miR-548ap-3p	0	0,601033056	0	0,422649731
hsa-miR-1277-5p	0	2,022427371	0	0,422649731
hsa-miR-23a-5p	0	1,685356143	0	0,422649731
hsa-miR-548at-5p	0	1,685356143	0	0,422649731
hsa-miR-144-3p	27,19033106	471,2343446	0,057700232	0,428278496
hsa-miR-92a-3p	42989,00673	61492,03073	0,69909883	0,428590782
hsa-miR-144-5p	3,050661786	44,34718336	0,068790429	0,429643857
hsa-miR-629-5p	165,1074625	62,4625151	2,643304744	0,432129
hsa-miR-378g	1,591554153	3,127835476	0,508835636	0,440676131
hsa-miR-365b-3p	6,100957585	14,3826233	0,424189486	0,4444598
hsa-miR-30a-5p	119,9033013	37,99376714	3,155867667	0,449275464
hsa-miR-107	219,6648407	24,41672358	8,996491278	0,454680174
hsa-miR-338-3p	45,76578072	8,874204205	5,157170115	0,455853758
hsa-miR-941	191,1657782	276,5920814	0,691146967	0,459724365
hsa-miR-103a-3p	2292,382416	2948,294901	0,777528196	0,459761401
hsa-miR-30b-3p	4,292439864	7,677065268	0,559125097	0,4607242

hsa-miR-1291	43,6235848	18,56630608	2,349610343	0,460728377
hsa-miR-30d-5p	12622,3	16619,43565	0,75949029	0,465992338
hsa-miR-30c-5p	7821,618301	9604,620736	0,814359933	0,466758447
hsa-miR-30e-3p	1312,005178	1039,169279	1,262551929	0,467469039
hsa-miR-616-5p	9,700431211	6,736497397	1,439981438	0,4678242
hsa-let-7d-5p	2996,209381	3931,702172	0,762064177	0,478309563
hsa-miR-200c-3p	70,48353153	98,00258923	0,719200708	0,492360766
hsa-miR-378a-5p	10,15850608	13,3738732	0,759578465	0,50225093
hsa-miR-628-3p	17,98968426	28,45418756	0,632233277	0,505548637
hsa-miR-486-3p	201,0407314	157,6012307	1,275629198	0,506617274
hsa-miR-423-3p	4689,645325	6163,113609	0,760921447	0,511352938
hsa-miR-548ar-5p	3,231403762	1,682892556	1,920148586	0,513071561
hsa-miR-362-5p	43,52735987	27,92626391	1,558653174	0,514507394
hsa-miR-128	578,7062901	462,7627791	1,250546319	0,517976564
hsa-miR-4454	1,663997338	4,091951952	0,406651241	0,51893055
hsa-miR-192-5p	1453,510447	1005,788795	1,4451448	0,523247124
hsa-miR-532-3p	14,10116987	7,092190056	1,988267341	0,530786371
hsa-miR-3615	40,68195378	51,1792515	0,794891535	0,541083577
hsa-miR-644b-3p	13,8842063	9,017959421	1,539617296	0,543661065
hsa-miR-577	300,0760146	184,449475	1,62687378	0,546362885
hsa-miR-3184-5p	40,90513727	23,32008256	1,754073433	0,54921768
hsa-miR-548e	9,513469309	5,529504112	1,720492311	0,552122523
hsa-miR-320b	4,973698224	6,47253557	0,768431192	0,556496207
hsa-miR-3613-3p	15,56613144	21,2653395	0,731995435	0,556899669
hsa-let-7a-5p	44119,09839	54790,20359	0,805236986	0,560609683
hsa-miR-106a-5p	14,87901914	10,65375477	1,396598614	0,562346785
hsa-miR-324-5p	45,15513562	27,14807268	1,663290656	0,569463717
hsa-miR-200b-3p	56,23784128	28,43802972	1,977557582	0,570967864
hsa-miR-16-2-3p	464,0613397	495,9940027	0,935618853	0,578382384
hsa-miR-548w	2,990292465	1,803099167	1,658418195	0,583885046
hsa-miR-618	1,856813178	5,769917334	0,321809321	0,584725742
hsa-miR-24-2-5p	15,08390884	19,70018997	0,765673269	0,585584235
hsa-miR-574-5p	17,00109134	20,64568501	0,823469472	0,585843383
hsa-miR-18a-5p	20,49775413	27,21734218	0,753113732	0,585869178
hsa-miR-1270	4,34073532	2,644545445	1,641391843	0,587190048
hsa-miR-502-3p	150,4176011	205,5477997	0,731788914	0,587654083
hsa-miR-21-3p	322,5443687	222,1647878	1,45182489	0,58921747
hsa-miR-25-5p	57,74926838	75,70495102	0,762820233	0,591408484
hsa-miR-5001-3p	1,802663782	0,601033056	2,999275606	0,591801012
hsa-miR-511	2,495996006	0,841446278	2,966316534	0,594072875
hsa-miR-589-5p	103,0203867	130,2242645	0,791099777	0,594735261
hsa-miR-31-5p	15,45197871	26,09222537	0,592206241	0,595992288
hsa-miR-100-5p	49,84528103	80,49488309	0,619235399	0,596721346

hsa-miR-342-3p	5530,425338	6787,220225	0,814829217	0,602612271
hsa-miR-92b-3p	129,8097183	178,0746847	0,728962225	0,604103642
hsa-miR-25-3p	3039,79089	3650,256299	0,832760947	0,608693437
hsa-miR-301a-5p	2,387331229	3,849075143	0,620235028	0,609235614
hsa-miR-193b-3p	0,831998669	2,283925611	0,364284487	0,610914949
hsa-miR-1271-5p	320,4120489	468,4275604	0,684016219	0,612221739
hsa-miR-642a-5p	1,591554153	4,327438	0,367782081	0,61310535
hsa-miR-331-3p	182,1169697	225,140101	0,808905072	0,627792623
hsa-miR-378a-3p	1300,241213	1487,872234	0,873893055	0,629676071
hsa-miR-215	8,651468973	4,808264445	1,799291423	0,633187172
hsa-miR-296-5p	5,136512397	7,335066866	0,700267972	0,639743748
hsa-miR-576-5p	33,5986232	22,72151309	1,478714163	0,640656522
hsa-miR-199b-5p	82,54056033	116,496075	0,708526535	0,643114358
hsa-miR-3074-5p	8,434139418	5,821941718	1,448681527	0,643951373
hsa-miR-103a-2-5p	6,197548498	4,930934643	1,256870948	0,644154317
hsa-miR-10a-5p	338,3519756	407,8609626	0,829576759	0,650585802
hsa-miR-185-3p	1,591554153	3,726404945	0,427101771	0,650895267
hsa-miR-1285-3p	17,52575545	16,52772087	1,060385494	0,653000467
hsa-miR-500a-3p	131,8425175	158,7403308	0,830554635	0,657155751
hsa-miR-23b-5p	7,487622033	10,87801015	0,688326443	0,658851552
hsa-miR-576-3p	6,31828714	3,971745341	1,590808724	0,660324953
hsa-miR-204-5p	1,326295127	0,601033056	2,206692485	0,667807038
hsa-miR-27b-3p	5005,175501	3763,090035	1,330070621	0,669010207
hsa-miR-106a-3p	1,247998003	0,601033056	2,076421574	0,686398782
hsa-miR-181c-5p	17,91175312	22,41784481	0,798995321	0,687228921
hsa-miR-659-5p	2,990292465	2,043512389	1,463310172	0,692441137
hsa-miR-497-5p	1,247998003	2,524338834	0,494386089	0,694796908
hsa-miR-30c-1-3p	19,95516221	24,98051377	0,798829135	0,698515778
hsa-miR-330-5p	11,7259125	9,114617428	1,286495302	0,70394933
hsa-miR-766-3p	46,93950561	57,49571392	0,816400083	0,704229591
hsa-miR-96-5p	38,42780104	48,95227745	0,785005378	0,705361222
hsa-miR-582-5p	7,144065884	12,74190078	0,560675052	0,706221076
hsa-miR-5196-3p	4,774662458	2,623460427	1,819986461	0,707969299
hsa-miR-363-3p	743,5861828	997,3473019	0,745563939	0,708921361
hsa-miR-664-3p	371,6955837	299,374386	1,241574433	0,715448176
hsa-miR-223-3p	1084,201819	1475,512465	0,734796788	0,72081111
hsa-miR-1296	0,970665114	1,803099167	0,538331519	0,723741903
hsa-miR-145-3p	2,652590254	3,728868532	0,71136599	0,726513835
hsa-miR-24-3p	2451,246597	2756,297624	0,889325803	0,733452289
hsa-miR-671-3p	65,60496028	72,42596957	0,905820946	0,734574797
hsa-miR-140-3p	1476,794698	1323,220185	1,116061193	0,735261937
hsa-miR-574-3p	75,14769928	91,21653103	0,823838601	0,744121189
hsa-miR-27a-5p	30,34270572	35,09402695	0,864611684	0,751036986

hsa-let-7d-3p	278,7971155	224,1496833	1,243798838	0,751635524
hsa-let-7f-1-3p	10,16436002	7,455273477	1,36337856	0,752309006
hsa-miR-148b-3p	797,5936494	740,9643547	1,076426476	0,762987564
hsa-miR-23b-3p	194,7923256	146,1931224	1,332431529	0,76908612
hsa-miR-874	91,89670342	111,3742879	0,825115968	0,771112094
hsa-miR-186-5p	8374,719622	7878,474117	1,062987515	0,77417862
hsa-let-7c	24,94678824	29,11463576	0,856846998	0,779327391
hsa-miR-1299	1,326295127	0,841446278	1,576208918	0,78674845
hsa-miR-148a-5p	63,56081921	55,86238216	1,137810755	0,79336578
hsa-miR-1248	21,25694363	29,05029344	0,73172905	0,795146539
hsa-miR-708-5p	22,6026305	16,0022608	1,412464825	0,801079367
hsa-miR-339-5p	158,3804939	134,4996781	1,177552958	0,802434571
hsa-miR-548o-3p	6,31828714	7,936099921	0,796145109	0,808240515
hsa-miR-424-5p	5,781549164	4,447644612	1,299912576	0,812874954
hsa-miR-99a-5p	139,026829	176,861966	0,786075334	0,813792252
hsa-miR-425-3p	27,17789121	25,30526707	1,074001359	0,8141894
hsa-miR-148a-3p	7888,058909	8665,346771	0,910299278	0,81722751
hsa-let-7f-5p	54269,42503	56867,83744	0,954307874	0,818035767
hsa-miR-1246	415,9173719	324,6353085	1,281183411	0,822816288
hsa-miR-10b-5p	6,794655795	5,051141254	1,345172398	0,8265329
hsa-miR-3173-5p	6,667697228	7,891466298	0,844925008	0,829720128
hsa-miR-197-3p	273,9562298	233,8867079	1,171320219	0,831626921
hsa-miR-499a-5p	48,00749361	46,43424211	1,033881279	0,835488209
hsa-miR-3607-3p	1,856813178	1,322272722	1,404258854	0,836418374
hsa-miR-223-5p	78,31470971	70,35289414	1,113169695	0,839031781
hsa-miR-532-5p	146,8792288	133,9643637	1,096405229	0,851141327
hsa-miR-30b-5p	5939,340621	5677,602838	1,046100051	0,858770743
hsa-miR-26b-3p	68,99332427	70,65764968	0,97644522	0,859012063
hsa-miR-143-3p	114,6650741	131,9131715	0,869246587	0,861119257
hsa-miR-33a-3p	1,591554153	1,202066111	1,324015491	0,863212988
hsa-miR-152	138,6499791	144,859619	0,957133396	0,866822028
hsa-miR-132-3p	5,7694753	4,808264445	1,199908068	0,877873528
hsa-miR-3605-3p	1,386664448	1,0818595	1,281741712	0,878363992
hsa-miR-1179	1,386664448	1,0818595	1,281741712	0,878363992
hsa-miR-766-5p	1,326295127	1,685356143	0,786952439	0,882435425
hsa-miR-1307-5p	30,78321877	29,61901081	1,039306105	0,886953557
hsa-miR-221-5p	524,6805261	561,7434681	0,934021588	0,887911092
hsa-miR-196a-5p	6,100957585	7,214860254	0,845609945	0,891417785
hsa-miR-17-3p	47,24208419	44,93973839	1,051231847	0,893564256
hsa-miR-23a-3p	589,865829	628,5400138	0,938469813	0,894328632
hsa-miR-331-5p	17,76101281	16,72350047	1,062039185	0,896120966
hsa-miR-151a-5p	6515,320024	6075,931323	1,072316272	0,89977081
hsa-miR-92a-1-5p	38,68647415	41,32230939	0,93621278	0,91011797

hsa-miR-550a-5p	2,079996672	1,803099167	1,153567541	0,929051414
hsa-miR-195-3p	0,693332224	0,601033056	1,153567541	0,929051414
hsa-miR-200b-5p	0,693332224	0,601033056	1,153567541	0,929051414
hsa-miR-184	17,11012211	15,70735961	1,089306066	0,929594965
hsa-miR-28-5p	788,7808302	757,9849998	1,040628549	0,933766106
hsa-miR-505-3p	75,81761575	79,46504798	0,95410017	0,933834933
hsa-miR-95	5,311400434	5,769917334	0,920533194	0,944829909
hsa-miR-4775	0,970665114	1,0818595	0,897219199	0,945983851
hsa-miR-183-5p	11,00843255	10,27944068	1,070917465	0,947994403
hsa-miR-145-5p	4,774662458	5,051141254	0,945264093	0,961405711
hsa-miR-93-5p	492,9943294	485,9226596	1,014553077	0,966131091
hsa-miR-196b-5p	3,713626356	3,849075143	0,964810044	0,974429079
hsa-let-7b-3p	49,5979498	50,70965573	0,978077037	0,976044427
hsa-miR-221-3p	1647,030252	1625,66785	1,013140693	0,976529493
hsa-miR-151a-3p	1201,647172	1180,131061	1,018231968	0,976769029
hsa-miR-345-5p	84,09589289	84,78015106	0,991929029	0,987391377
hsa-miR-301a-3p	2,435626686	2,404132222	1,013100138	0,990774689
hsa-miR-30d-3p	36,13669474	36,08661921	1,001387648	0,991200053
hsa-miR-148b-5p	43,16624191	43,13663923	1,000686254	0,992177799
hsa-miR-195-5p	77,64515923	77,52558436	1,001542392	0,996549169
hsa-miR-501-5p	1,326295127	1,322272722	1,003042039	0,998481294
