# Supplementary material for: TGF-β/SMAD Pathway Is Modulated by miR-26b-5p: Another Piece in the Puzzle of Chronic Lymphocytic Leukemia Progression
Source: Cancers (Basel). 2022 Mar 25;14(7):1676. doi: 10.3390/cancers14071676 (PMC8997107; doi:10.3390/cancers14071676)
Supplement: Supplementary file 1 [file cancers-14-01676-s001.zip › Supplementary Table S2, Primer sequences.pdf]

Supplementary Table S2: Primer sequences

Primer Name	Primer sequences (5' to 3')	
P21	**Fw** CATGTGGACCTGTCACTGTCTTGT	**Rev** GAAGATCAGCCGGCGTTTG
KLF10	**Fw** CTCACATTGCCGCACCTTTC	**Rev** TGCTTTCATTGGGCAGGTCT
SMAD 2	**Fw** GCTGGCCTGATCTTCACAGT	**Rev** CCAGAGGCGGAAGTTCTGTT
SMAD 3	**Fw** AACGGGCAGGAGGAGAAATG	**Rev** CTGGGGATGGTGATGCACTT
SMAD 4	**Fw** CCCATCCCGGACATTACTGG	**Rev** TAGGGCAGCTTGAAGGAACC
SMAD 7	**Fw** GGACCAAACGATCTGCGCTC	**Rev** GGGATGGTGGTGACCTTTGG
U6	**Fw** CTCGCT TCGGCAGCACA	**Rev** AACGTCTCACGAATTTGCGT
qGAPDH	**Fw** TCGGAGTCAACGGATTTG	**Rev** CCTGGAAGATGGTGATGG
GAPDH	**Fw** GGTGCTGAGTATGTCGTGGA	**Rev** ATGCCAGTGAGCTTCCCGTT
C-myc	**Fw** CTTTGTGTGCCCCGCTCCAG	**Rev** GCGCTCAGATCCTGCAGGTA
SL miR-26b-5p	GTCGTATCCAGTGCAGGGTCCGAGGTATTCGCACTGGATACGACAACCTA	
SL miR-106b-5p	GTCGTATCCAGTGCAGGGTCCGAGGTATTCGCACTGGATACGACATCTGC	
SL miR-142-5p	GTCGTATCCAGTGCAGGGTCCGAGGTATTCGCACTGGATACGACAGTAGT	
SL miR-7-5p	GTCGTATCCAGTGCAGGGTCCGAGGTATTCGCACTGGATACGACAACAAC	
SL miR-let-7-g	GTCGTATCCAGTGCAGGGTCCGAGGTATTCGCACTGGATACGACAACTGT	
SL miR-19a-3p	GTCGTATCCAGTGCAGGGTCCGAGGTATTCGCACTGGATACGACAGTTTT	
SL miR-374b-5p	GTCGTATCCAGTGCAGGGTCCGAGGTATTCGCACTGGATACGACACTTAG	
SL miR-146a-5p	GTCGTATCCAGTGCAGGGTCCGAGGTATTCGCACTGGATACGACAACCCA	
Uni Rev	GTGCAGGGTCCGAGGT	
miR-106b-5p	**Fw** GACCGCATAAAGTGCTGACAGTG	
miR-142-5p	**Fw** AGGCTCGCATAAAGTAGAAAGCACT	
miR-7-5p	**Fw** ACGGCGTGGAAGACTAGTGATTTT	
miR-let-7-g	**Fw** CGTGCCTGAGGTAGTAGTTTGTAC	
miR-19a-3p	**Fw** CAGCCATGTGCAAATCTATGCAAA	
miR-374b-5p	**Fw** GCGGCATATAATACAACCTGCTAA	
miR-146a-5p	**Fw** GCCGAGTGAGAACTGAATTCCATG	
miR-26b-5p	**Fw** GGCGCGTTCAAGTAATTCAGGATAG	
